# Supplementary material for: Maternal Thyroid Supplementation Prevents Autistic-relevant Social Behavior and Hypothalamic Oxytocin Depletion Produced by Developmental Exposure to Environmental Toxicants
Source: bioRxiv. 2025 Jul 31:2025.07.28.667293. Preprint. [Version 1] doi: 10.1101/2025.07.28.667293 (PMC13255000; doi:10.1101/2025.07.28.667293)
Supplement: Supplement 1 [file media-1.pdf]

Maternal Thyroid Supplementation Prevents Autistic-relevant Social Behavior and Hypothalamic Oxytocin Depletion Produced by Developmental Exposure to Environmental Toxicants

Elena V. Kozlova[1,2], Maximillian E. Denys[1,3], Anthony E. Bishay[1], Crystal N. Luna[1], Meri De Angelis[4,5], Luis Campoy[1], Amna Habbal[1], Artha A. Lam[1], Naran Luvsanravdan[1] , Anastasia Ghilenschi Colton[1], David Carter[6], Timo Müller[4], Karl-Werner Schramm[7,8] and Margarita C. Currás-Collazo[1]*

Orcid IDs: E.V.K.: 0000-0002-4691-6618; M.E.D.: 0000-0002-7423-6265; A.E.B.: 0000-0002-6057-3770; C.N.L.: 0009-0003-3003-0665; A.A.L.: 0009-0001-1023-5204; N.L.: 0009-0001-9881-5747; A.G.C.: 0009-0007-9975-9402; K.W.S.: 0000-0002-8945-4062; M.C.C-C.: 0000-0002-0189-4179

[1]Department of Molecular Cell and Systems, University of California, Riverside, CA
[2]Neuroscience Graduate Program, University of California, Riverside, CA
[3]University of California School of Medicine, Riverside, CA, USA
[4]Institute for Diabetes and Obesity, Helmholtz Munich, Neuherberg, Germany
[5]Institute of Experimental Genetics, Helmholtz Munich, Neuherberg, Germany
[6]Microscopy and Imaging Core Facility, College of Natural and Agricultural Sciences, University of California, Riverside, CA
[7]Molecular EXposomics, Helmholtz Munich, Neuherberg, Germany
[8]TUM, Wissenschaftszentrum Weihenstephan für Ernährung, Landnutzung und Umwelt, Department für Biowissenschaftliche Grundlagen, Freising, Germany

***Corresponding author**:
Dr. Margarita C. Curras-Collazo, Ph.D
Professor of Neuroscience
Department Molecular, Cell and Systems Biology
University of California, Riverside
Riverside, CA 92521
951-827-3960
mcur@ucr.edu

Supplementary Table 1

Supplementary Table 1. Dam Physiological Parameters during Gestation and Lactation

	PTU	VEH/CON	VEH/CON+T4	L-DE-71	L-DE-71+T4	H-DE-71	H-DE-71+T4
Maternal Parameters							
n[i]	7-12	7-17	4-12	7-12	6-15	5-13	7-14
**Gestational food intake** (GD15-0)							
Absolute (g/d)<br>Relative (g/d/pup)	7.67±0.74<br>0.96±0.12	7.00±0.67<br>0.94±0.09	8.88±0.70<br>1.45±0.18	7.42±0.37<br>0.99±0.07	6.98±0.33<br>1.16±0.11	6.28±0.35<br>0.97±0.15	8.87±1.45<br>1.76±0.37*^
**Postpartum food intake** (P1-P10)							
Absolute (g/d)<br>Relative (g/d/pup)	12.90±1.07<br>1.61±0.10*	15.59±1.02<br>2.10±0.13	12.13±0.95<br>2.09±0.18	14.94±1.15<br>1.98±0.14	13.02±0.71<br>2.09±0.14	11.59±0.43<br>1.82±0.18	13.36±1.24<br>2.28±0.22
**Gestational water intake** (GD15-P0)							
Absolute (mL/d)<br>Relative (mL/d/pup)	8.10±0.52<br>0.99±0.08	7.00±0.35<br>0.96±0.07	7.63±0.46<br>1.32±0.16	7.76±0.19<br>1.05±0.06	7.02±0.18<br>1.17±0.13	7.31±0.30<br>1.08±0.11	7.56±0.30<br>1.53±0.20*
**Postpartum water intake** (P1-P10)							
Absolute (mL/d)<br>Relative (mL/d/pup)	12.79±0.44*<br>1.58±0.09*	14.46±0.48<br>1.91±0.12	13.62±0.66<br>2.28±0.15	14.12±0.45<br>1.88±0.11	13.90±0.33<br>2.24±0.25	13.47±0.40<br>2.04±0.20	12.47±0.78<br>2.30±0.28
**Gestational weight gain** (GD15-P0)							
Absolute (g/d)<br>Relative (g/d/pup)<br>Abs. Percent Change (%)[ii]<br>Rel Percent Change (%)	1.85±0.21<br>0.23±0.02<br>41.19±6.09<br>4.94±0.46	1.90±0.11<br>0.22±0.03<br>52.80±4.28<br>6.74±0.67	1.58±0.20<br>0.26±0.03<br>44.38±6.50<br>7.41±1.04	1.58±0.14<br>0.22±0.02<br>50.34±4.72<br>6.98±0.75	1.59±0.17<br>0.24±0.03[b]<br>48.28±5.28<br>7.50±0.93	1.64±0.19<br>0.27±0.04<br>50.56±6.00<br>8.57±1.37	2.30±0.21<br>0.41±0.05**<br>52.91±3.51<br>8.94±0.73
**Postpartum weight gain** (P1-P10)							
Absolute (g/d)<br>Relative (g/d/pup)<br>Abs. Percent Change (%)[iii]<br>Rel. Percent Change (%)	0.61±0.20<br>0.08±0.03<br>10.66±3.79<br>(*p*=0.12)<br>1.33±0.42<br>(*p*=0.13)	0.22±0.11<br>0.03±0.02<br>3.51±2.07<br>0.50±0.28	0.11±0.20<br>0.01±0.03<br>2.51±5.25<br>0.27±0.92	0.58±0.06<br>0.08±0.01<br>10.95±1.27<br>1.47±0.25	0.27±0.11<br>0.04±0.02<br>5.25±2.06<br>0.73±0.30	0.78±0.21[a]<br>0.09±0.02<br>17.22±4.24*[a]<br>1.95±0.43	0.58±0.13<br>0.08±0.02<br>11.21±2.12<br>1.56±0.28

**Number of Pups in Litter**	7.50±0.58	8.00±0.44	6.82±0.62	7.64±0.45	7.00±0.49	7.23±0.70	6.43±0.68
**Secondary Sex Ratio** (males/males+females)	0.63±0.04 (*p*=.055)	0.49±0.05	0.49±0.07	0.44±0.04	0.51±0.06	0.48±0.07	0.55±0.05

Maternal food intake was measured from GD15-P10. Relative food intake was normalized to total pups in litter (relative). Gestational weight gain was calculated as difference in weight from pre-pregnancy to late gestation (GD 15-P0). Relative weight gain was normalized to pre-pregnancy weight (initial) and to total pups in litter (relative). Gestational weight gain relative to initial weight was calculated as percent change from weight at GD 2-3 (whichever was 18d prior to birth) until birth (usually GD19). Data are expressed as mean±s.e.m.

[i]Indicates number of dams/treatment group

[ii]Change vs initial weight at 18d before pup birth

[iii]Mean change per day vs initial weight at P1

*Statistical difference vs VEH/CON, *$p<0.05$

^Statistical difference with corresponding mT4 group, ^$p<0.05$

[a]Statistical difference vs VEH/CON+mT4, [a]$p<0.05$

[b]Statistical difference vs H-DE-71+mT4, [b]$p<0.05$

Supplementary Statistical Information for Supplementary Table 1

Gestational food intake was measured from GD15 until birth (P0). Gestational absolute food intake differed based on T4 treatment (Two-Way ANOVA: *Treatment Effect* $F_{(1,47)}=5.29$, $p=.025$), but there were no differences on post-hoc comparisons. No differences were observed across exposure or interaction (*Exposure Effect* $F_{(2,47)}=0.54$, ns; *Treatment x Exposure Interaction* $F_{(2,47)}=2.67$, ns). No difference was seen between PTU and VEH/CON groups (Independent *t*-test: t=0.67, *ns*). Gestational relative food intake, defined as absolute food intake per number of pups per dam, differed based on T4 treatment (*Treatment Effect* $F_{(1,46)}=11.45$, $p=.002$), with H-DE-71+T4 dams showing significantly greater relative food intake compared to VEH/CON ($p=.018$), and H-DE-71 ($p=.037$). No differences were observed across exposure or interaction (Two-Way ANOVA: *Exposure Effect* $F_{(2,46)}=1.45$, *ns*; *Treatment x Exposure Interaction* $F_{(2,46)}=1.69$, ns). No difference was seen between PTU and VEH/CON groups (Independent *t*-test: t=0.10, *ns*).

There were no group differences in postpartum absolute food intake from P1-10 (Two-Way ANOVA: *Treatment Effect* $F_{(1,50)}=2.01$, *ns*; *Exposure Effect* $F_{(2,50)}=1.24$, ns; *Treatment x Exposure Interaction* $F_{(2,50)}=3.15$, *ns*). No difference was seen between PTU and VEH/CON groups (Independent *t*-test: t=1.75, *ns*). Likewise, postpartum relative food intake was not different across groups (Two-Way ANOVA: *Treatment Effect* $F_{(1,50)}=1.94$, ns; *Exposure Effect* $F_{(2,50)}=0.01$, *ns*; *Treatment x Exposure* $F_{(2,50)}=1.01$, *ns*). Relative food intake was higher in VEH/CON compared to PTU (Independent *t*-test: t=2.69, $p=.016$) (**Supplementary Table 1**).

Gestational water intake was also measured from GD15 until birth (P0). Gestational absolute water intake was not different across groups (Two-Way ANOVA: *Treatment Effect* $F_{(1,53)}=0.04$, *ns*; *Exposure Effect* $F_{(2,53)}=0.08$, *ns*; *Treatment x Exposure* $F_{(2,53)}=2.70$, *ns*). No difference was seen between PTU and VEH/CON groups (Independent *t*-test: t=1.84, *ns*). Gestational relative water intake, defined as absolute water intake per number of pups per dam, differed by T4 treatment (Two-Way ANOVA: *Treatment Effect* $F_{(1,52)}=8.94$, $p=.004$), with H-DE-71-T4 dams showing significantly greater water intake compared to VEH/CON ($p=.025$). However, relative gestational water intake did not differ by exposure or interaction (*Exposure Effect* $F_{(2,52)}=1.36$, ns; *Treatment x Exposure Interaction* $F_{(2,52)}=0.89$, ns). No difference was seen between PTU and VEH/CON groups (Independent *t*-test: t=0.35, *ns*).

Similarly, there were no group differences in postpartum absolute water intake from P1-10 (Two-Way ANOVA: *Treatment Effect* $F_{(1,48)}=2.39$, *ns*; *Exposure Effect* $F_{(2,48)}=2.60$, *ns*; *Treatment x Exposure Interaction* $F_{(2,48)}=0.307$, *ns*). Absolute water intake was higher in VEH/CON compared to PTU (Independent *t*-test: t=2.53,

p=.023). Postpartum relative water intake differed by T4 treatment (Two-Way ANOVA: *Treatment Effect* $F_{(1,48)}$=4.06, *p*=.050, but no differences were seen on post hoc analysis. Similarly, relative water intake did not differ by exposure or interaction (*Exposure Effect* $F_{(2,48)}$=0.166, ns; *Treatment x Exposure Interaction* $F_{(2,48)}$=0.05, *ns*). Relative water intake was higher in VEH/CON compared to PTU (Independent *t*-test: t=2.23, *p*=.042)(**Supplementary Table 1**).

Daily gestational weight gain was also monitored from GD7 to birth (P0). A difference in gestational absolute weight gain was observed based on the interaction of treatment and exposure (*Treatment x Exposure Interaction* $F_{(2,64)}$=3.99, *p*=.023). However, no differences were observed on Holm-Sidak post-hoc test.No differences were seen across treatment or exposure effects (Two-Way ANOVA; *Treatment Effect* $F_{(1,64)}$=0.66, *ns*; *Exposure Effect* $F_{(2,64)}$=2.41, *ns*). No difference was seen in gestational absolute weight gain between PTU and VEH/CON groups (Independent *t*-test: t=0.24, *ns*). Similarly, gestational relative weight gain differed across treatment (*Treatment Effect* $F_{(1,64)}$=5.06, *p*=.028) and exposure effect (*Exposure Effect* $F_{(2,64)}$=5.36, *p*=.007 with H-DE-71+T4 showing greater gestational relative weight gain compared to VEH/CON (*p*=.003), and L-DE-71+T4 dams (*p*=.013). No interaction effects were seen for relative weight gain (*Treatment x Exposure Interaction* $F_{(2,64)}$=1.39, *ns*). No difference was seen in gestational relative weight gain between PTU and VEH/CON groups (Independent *t*-test: t=0.50, *ns*). Percent change in absolute weight gain was not different across groups (*Treatment Effect* $F_{(1,64)}$=0.44, *ns*; *Exposure Effect* $F_{(2,64)}$=0.22, *ns*; *Treatment x Exposure Interaction* $F_{(2,64)}$=0.58, *ns*). No difference was seen in percent change in absolute weight gain between PTU and VEH/CON groups (Independent *t*-test: t=1.60, *ns*). Similarly, percent change in relative weight gain was not different across groups (*Treatment Effect* $F_{(1,63)}$=0.48, *ns*; *Exposure Effect* $F_{(2,63)}$=2.05, *ns*; *Treatment x Exposure Interaction* $F_{(2,63)}$=0.01, *ns*). No difference was seen in percent change in relative weight gain between PTU and VEH/CON groups (Independent *t*-test: t=1.94, *ns*).

Postpartum absolute weight gain from P1-10 differed across exposure effect (*Exposure Effect* $F_{(2,29)}$=6.98, *p*=.003), with H-DE-71 dams showing greater weight gain compared to VEH/CON+T4 (*p*=.038). However, no differences were observed across treatment or interaction (*Treatment Effect* $F_{(1,29)}$=3.66, *ns*; *Treatment x Exposure Interaction* $F_{(2,29)}$=0.28, ns). No difference was seen in postpartum absolute weight gain between PTU and VEH/CON groups (Independent *t*-test: t=1.73, *ns*). Similarly, postpartum relative weight gain differed across exposure effect (*Exposure Effect* $F_{(2,29)}$=5.27, *p*=.011), but post-hoc analysis showed no differences across groups. Postpartum Relative weight gain did not differ by treatment or interaction (*Treatment Effect* $F_{(1,29)}$=1.72, *ns*; *Treatment x Exposure Interaction* $F_{(2,29)}$=0.39, ns). No difference was seen in postpartum relative weight gain between PTU and VEH/CON groups (Independent *t*-test: t=0.50, *ns*). Percent change in absolute weight gain was different across exposure groups (*Exposure Effect* $F_{(2,29)}$=8.06, *p*=.002), with H-DE-71 showing a greater absolute percent change compared to VEH/CON (*p*=.016) and VEH/CON+T4 (*p*=.027). However, percent change in absolute weight gain did not differ by treatment or interaction (*Treatment Effect* $F_{(1,29)}$=3.66, ns; *Treatment x Exposure Interaction* $F_{(2,29)}$=0.51, *ns*). No difference was seen in percent change in absolute weight gain between PTU and VEH/CON groups (Independent *t*-test: t=1.66, *ns*). Percent change in relative weight gain differed by exposure effect (*Exposure Effect* $F_{(2,29)}$=5.77, *p*=.008), but no differences were seen on post-hoc analysis. Percent change in relative weight gain did not differ based on treatment or interaction (*Treatment Effect* $F_{(1,29)}$=2.01, *ns*; *Treatment x Exposure Interaction* $F_{(2,29)}$=0.230, *ns*). No difference was seen in percent change in relative weight gain between PTU and VEH/CON groups (Independent *t*-test: t=1.64, *ns*).

There were no exposure or treatment effects on secondary sex ratio or the average number of pups per litter.

Supplementary Figure 1

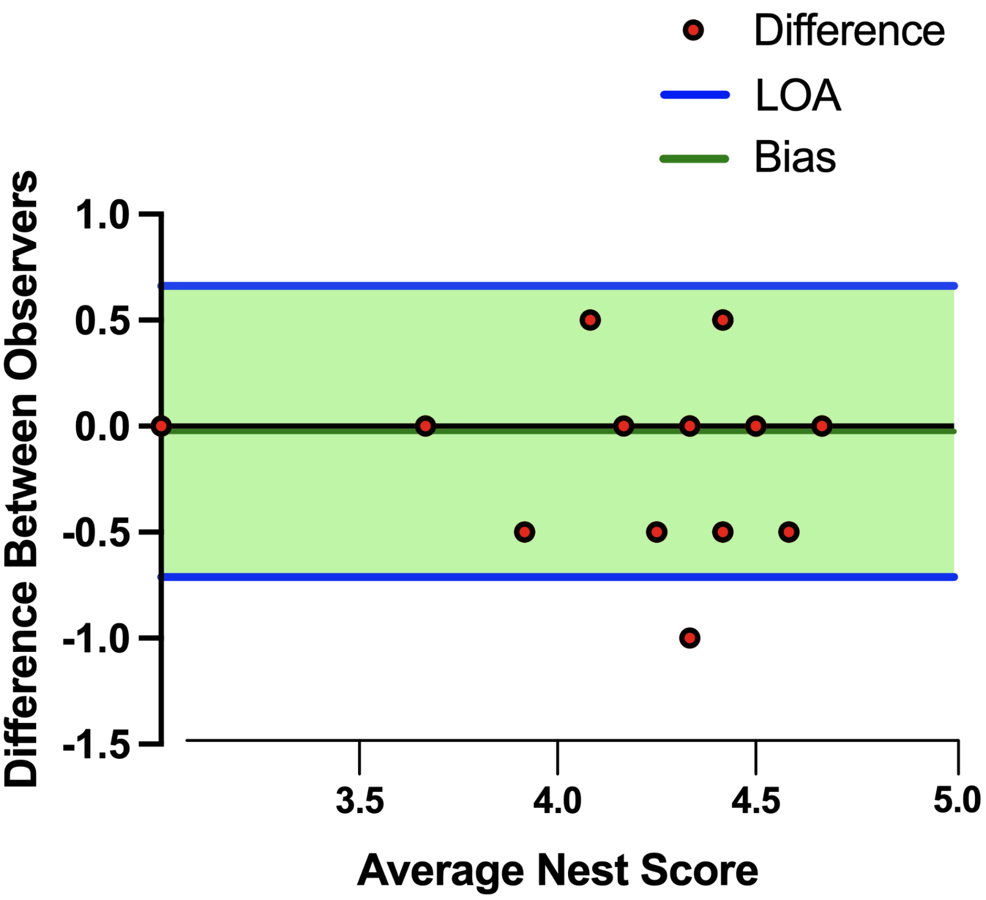

Supplementary Figure 1. Bland-Altman plot for nest scores (Related to Figure 1). Bland-Altman bias plot (mean±s.d.) was used to test the validity and reproducibility between two independent observers blind to exposure group. Analysis revealed a very small mean of the differences between observer scores (Bias, -0.023±0.35) and a precision measured as limits of agreement (LOA), average difference ± 1.96 standard deviation of the difference, of -0.71 to 0.66, indicating negligible skewing by either observer.

Supplementary Table 2. Elution parameters for the analysis of THs (Related to Figure 2).

**TIME (min)**	**PUMB B (%)**
0.1	5
3	5
4	30
5.5	35
6.5	35
7.0	40
8.0	100
10.0	100
10.2	5
13.2	5

Supplementary Table 3. Optimized MS/MS parameters for THs (Related to Figure 2). For each compound, ion-transitions are shown as m/z for the parent ion and two product ions (for quantification (q) and confirmation (c)). Compound optimized values for retention time (t_R), declustering potential (DP), collision energy (CE), and collision cell exit potential (CXP).

Compound	t_R (min)[a]	Parent ion (m/z)	Product ions (m/z)	DP (V)	CE (V)	CXP (V)
Target compounds						
T4	6.90	778	732(q)	75	40	20
			634 (c)	75	40	20
rT3	6.35	652	606 (q)	70	35	15
			508 (c)	70	35	15
T3	6.07	652	606 (q)	70	35	15
			508 (c)	70	35	15
3,3'-T2	5.67	526	480 (q)	65	40	20
			382 (c)	65	40	22
3,5-T2	5.34	526	480 (q)	65	35	20
			382 (c)	65	35	22
T1	5.11	400	256 (q)	70	35	20
			354 (c)	70	35	20
T_1AM	5.22	356	339 (q)	75	40	35
			212 (c)	75	40	25
Internal standards						
ML-T4	6.90	784	738	75	35	20
ML-rT3	6.35	658	612	70	35	15
ML-T3	5.67	658	612	70	35	15
ML-3,3'-T2	5.11	532	486	65	40	25
ML-T_1AM	5.22	362	345	75	40	35

[a] Retention time for the Shimadzu Nexera X2 LC.

Supplementary Figure 2

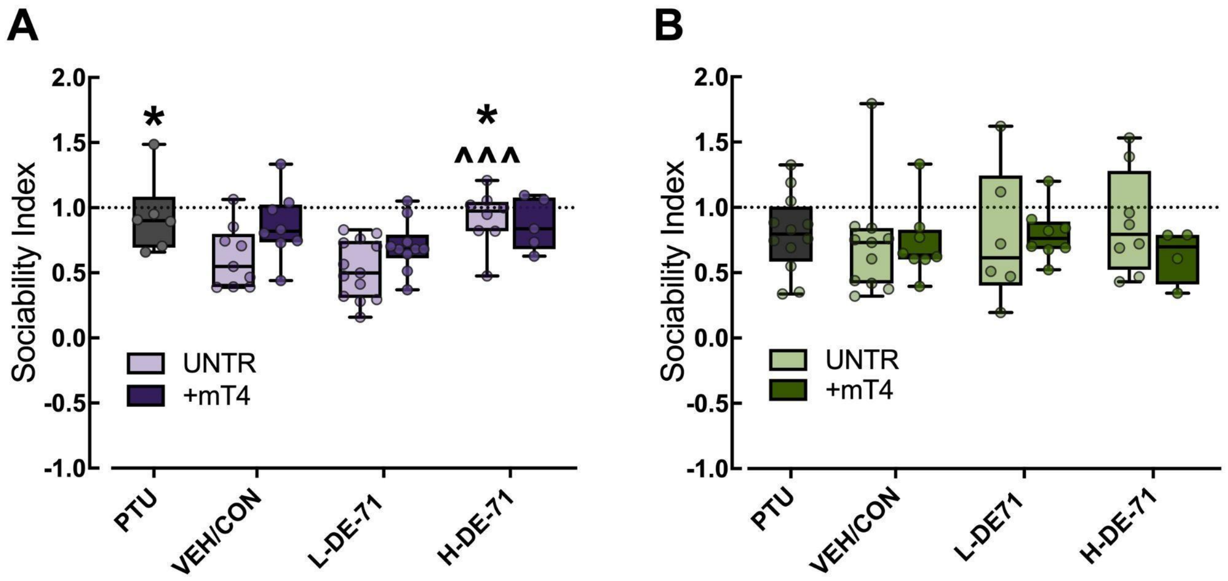

Supplementary Figure 2. Sociability Index (Related to Figure 4). (**A**) Sociability index in female offspring. (**B**) The sociability index in male offspring. *indicates a statistical difference vs VEH/CON in A (*p<.05). ^significant difference vs L-DE-71 in A (^^^p<.001). Values represent the means±s.e.m. n, 6-13/group (A) and 6-12/group (B). +mT4: maternal T4. UNTR: untreated

Supplementary Figure 3

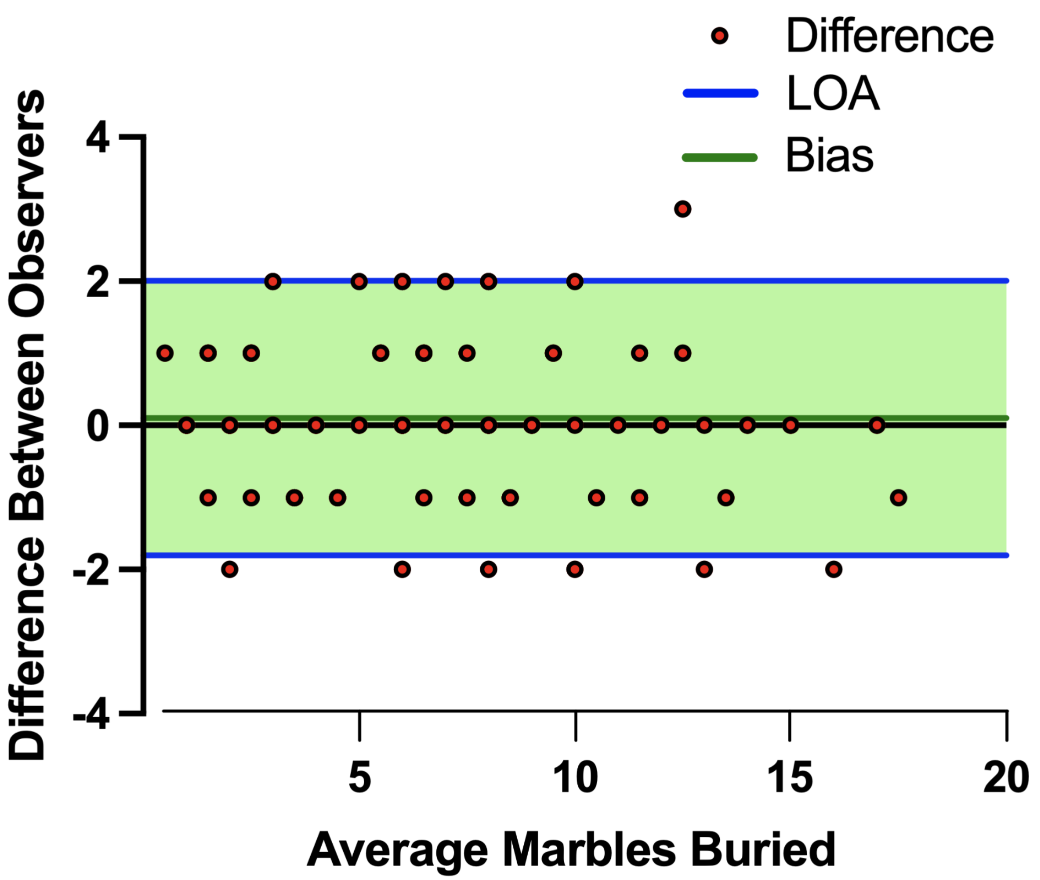

Supplementary Figure 3. Bland-Altman plot for marble (Related to Figure 4). Bland-Altman bias plot (mean±s.d.) was used to test the validity and reproducibility between two independent observers blind to exposure group. Analysis revealed a very small mean of the differences between observer scores (Bias, 0.10±0.97) and a precision measured as limits of agreement (LOA), average difference ± 1.96 standard deviation of the difference, of -1.8-2.2, indicating negligible skewing by either observer.

Supplementary Table 4: RT-PCR primer sequences and efficiencies and PCR products. (Related to Figure 5)

Target Gene	Gene Symbol	GenBank Accession Number	Primer Sequence Forward 5’-3’ Reverse 5’-3’	Exon target (Fwd/Rv)	E (%)	$Tm^{o}C$ (Fwd/Rv)	Product length (bp)	Anneal temp (°C)
Arginine vasopressin[a]	*Avp*	NM_009732.2	CTC AAC ACT ACG CTC TCC GC<br>CAG CAG ATG CTT GGT CCG A	1/1–2	98	60.8/57.9	173	55
Beta actin[a]	*Actb*	NM_007393	GATTACTGCTCTGGCTCCTAG<br>GACTCATCGTACTCCTGCTTG	5/6	99.6	55.0/54.4	147	60
Cyclic ADP ribose hydrolase	*Cd38*	NM_007646	TGC TCC TTT GAA TGT ACT CAG T<br>AGC ACT TTT CTG ACA TCT TCC T	1-2	100.9	57.4/57.6	110	60
Oxytocin[a]	*Oxt*	NM_011025.4	CCGAAGCAGCGTCTTTT<br>CTTGGCTTACTGGCTCTGAC	1/2	96.9	55.7/55.5	131	60
Thyrotropin-releasing hormone[b]	*Trh*	NM_009426.3	GATTCTGGAGCCTTGCAGAC<br>GGGGATACCAGTTAGCACGA	1/2	101.7	58.6/58.9	156	60

Abbreviations: *E* primer efficiency, *Tm* melting temperature, *bp* base pair
Primer sequences were obtained from: a: (Kozlova et al., 2022); b: (Swarnalatha & Abraham, n.d.)

Supplementary Figure 4

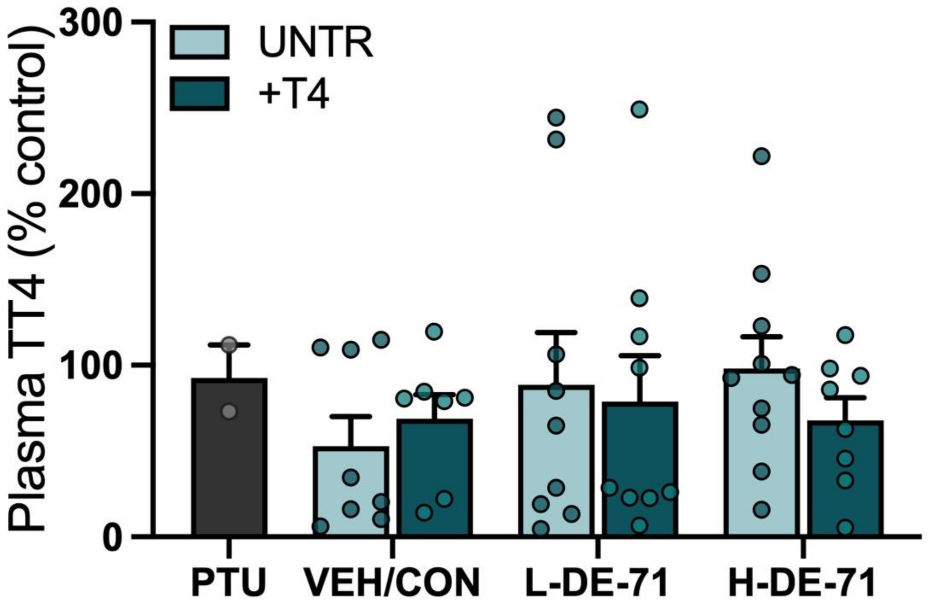

Supplementary Figure 4. (Related to Fig 1) Plasma total T4 in dams at P22. No statistical significance between groups and treatment. *n*, 2-10/group. UNTR: untreated

Supplementary Figure 5

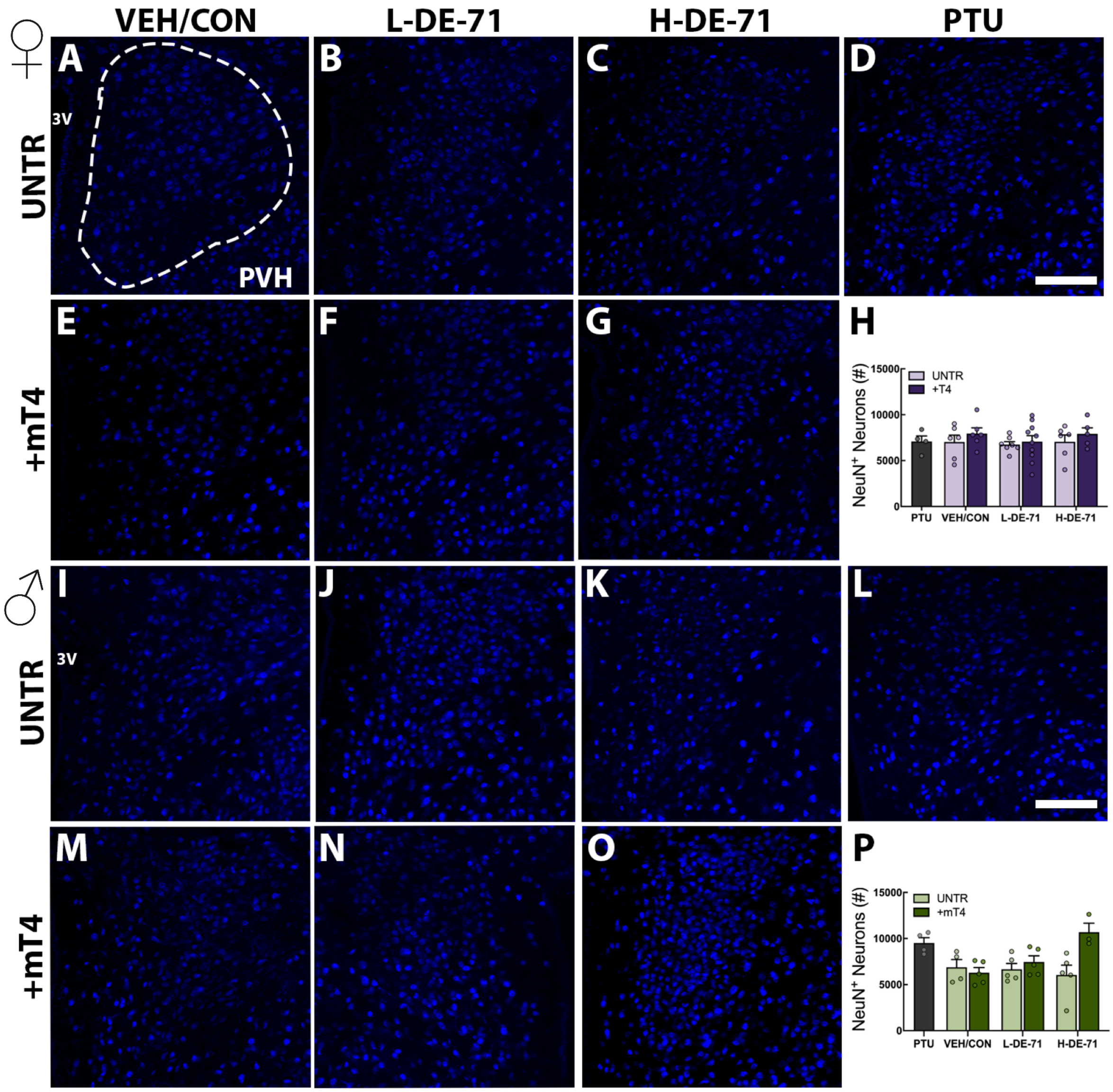

Supplementary Figure 5 (Related to Figure 6). NeuN+ neurons in PVH in females **(A-G)** and males **(I-O)**. Stereological quantification of Neun+ PVH neurons in females **(H)** and males **(P)**. The reduced OXT content in the PVH caused by L-DE-71 is not due to a reduction in global neuron number. *n*, 4-19/group (I); 4-10/group (H); 3-5/group (P) Scale bar, 100 microns

Supplementary Figure 6

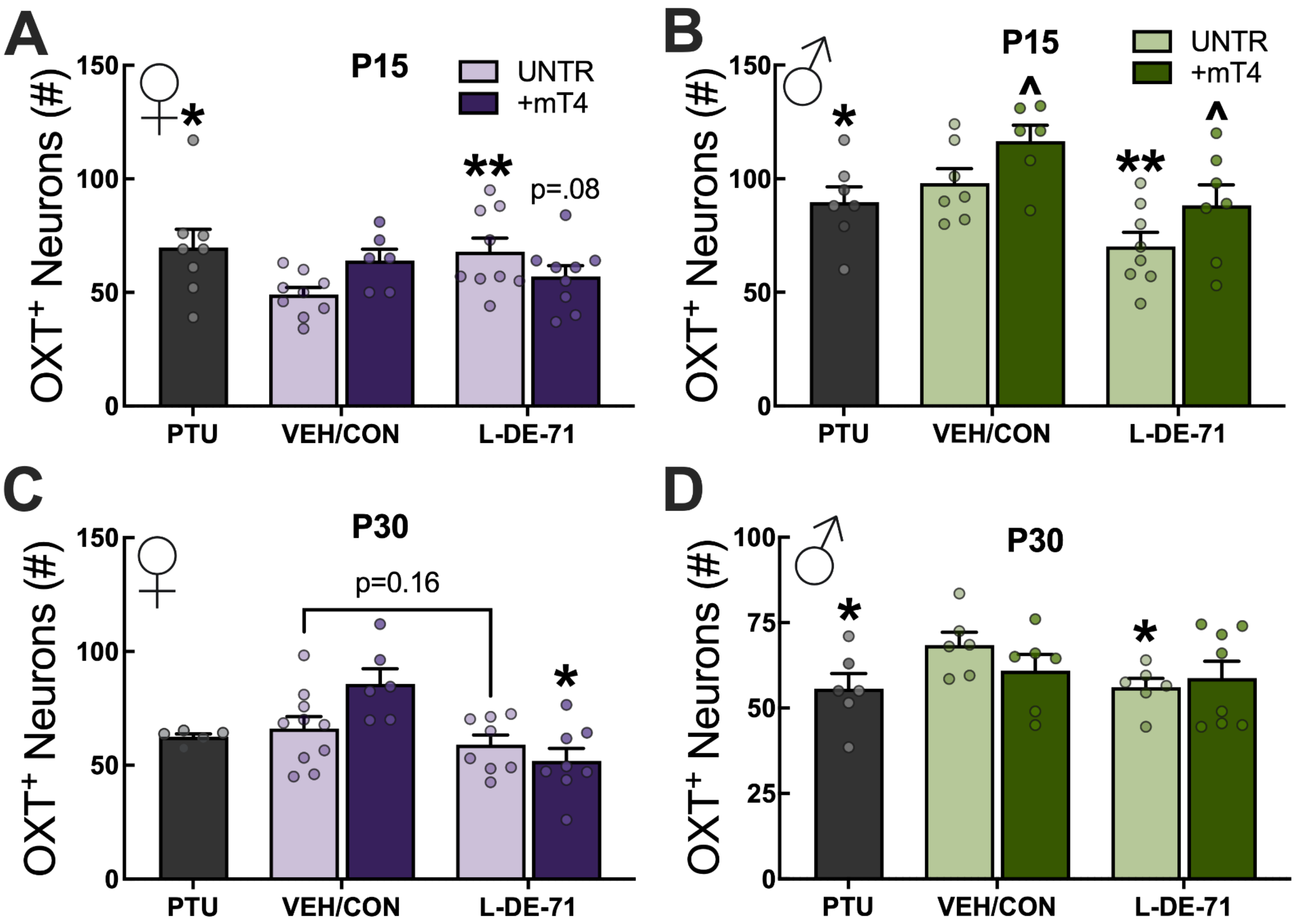

Supplementary Figure 6 (Related to Figures 8, 9). OXT+ neuron counts in PVH using HALO-AI digital image analysis in females at P15 (**A**), males at P15 (**B**), females at P30 (**C**), males at P30 (**D**). *n*, 6-9/group (A); 5-8/group (B); 5-10/group (C); 5-8/group (D)
